# TrawlerWeb: an online *de novo* motif discovery tool for next-generation sequencing datasets

**DOI:** 10.1186/s12864-018-4630-0

**Published:** 2018-04-05

**Authors:** Louis T. Dang, Markus Tondl, Man Ho H. Chiu, Jerico Revote, Benedict Paten, Vincent Tano, Alex Tokolyi, Florence Besse, Greg Quaife-Ryan, Helen Cumming, Mark J. Drvodelic, Michael P. Eichenlaub, Jeannette C. Hallab, Julian S. Stolper, Fernando J. Rossello, Marie A. Bogoyevitch, David A. Jans, Hieu T. Nim, Enzo R. Porrello, James E. Hudson, Mirana Ramialison

**Affiliations:** 1 Australian Regenerative Medicine Institute, Systems Biology Institute Australia, Monash University, Clayton, VIC Australia; 2 eResearch, Monash University, Clayton, VIC Australia; 3 UC Santa Cruz Genomics Institute, University of California, Santa Cruz, CA USA; 4 Department of Biochemistry and Molecular Biology, Bio21 Institute and Cell Signalling Research Laboratories, The University of Melbourne, Melbourne, VIC Australia; 5 CNRS, Inserm, Institute of Biology Valrose, Université Côte d’Azur, Parc Valrose, Nice, France; 6 School of Biomedical Sciences, The University of Queensland, QLD, Brisbane, Australia; 7 Centre for Innate Immunity and Infectious Diseases, Hudson Institute of Medical Research, Monash University, Clayton, VIC Australia; 8 Department of Biochemistry and Molecular Biology, Monash University, Clayton, VIC Australia; 9 Faculty of Information Technology, Monash University, Clayton, VIC Australia; 10 Murdoch Children’s Research Institute, The Royal Children’s Hospital, Parkville, VIC Australia; 11 Department of Physiology, School of Biomedical Sciences, The University of Melbourne, Parkville, VIC Australia

**Keywords:** Motif discovery, Transcription factor binding site, Motif conservation, Chromatin immunoprecipitation, Next generation sequencing

## Abstract

**Background:**

A strong focus of the post-genomic era is mining of the non-coding regulatory genome in order to unravel the function of regulatory elements that coordinate gene expression (Nat 489:57–74, 2012; Nat 507:462–70, 2014; Nat 507:455–61, 2014; Nat 518:317–30, 2015). Whole-genome approaches based on next-generation sequencing (NGS) have provided insight into the genomic location of regulatory elements throughout different cell types, organs and organisms. These technologies are now widespread and commonly used in laboratories from various fields of research. This highlights the need for fast and user-friendly software tools dedicated to extracting *cis*-regulatory information contained in these regulatory regions; for instance transcription factor binding site (TFBS) composition. Ideally, such tools should not require prior programming knowledge to ensure they are accessible for all users.

**Results:**

We present TrawlerWeb, a web-based version of the Trawler_standalone tool (Nat Methods 4:563–5, 2007; Nat Protoc 5:323–34, 2010), to allow for the identification of enriched motifs in DNA sequences obtained from next-generation sequencing experiments in order to predict their TFBS composition. TrawlerWeb is designed for online queries with standard options common to web-based motif discovery tools. In addition, TrawlerWeb provides three unique new features: 1) TrawlerWeb allows the input of BED files directly generated from NGS experiments, 2) it automatically generates an input-matched biologically relevant background, and 3) it displays resulting conservation scores for each instance of the motif found in the input sequences, which assists the researcher in prioritising the motifs to validate experimentally. Finally, to date, this web-based version of Trawler_standalone remains the fastest online *de novo* motif discovery tool compared to other popular web-based software, while generating predictions with high accuracy.

**Conclusions:**

TrawlerWeb provides users with a fast, simple and easy-to-use web interface for *de novo* motif discovery. This will assist in rapidly analysing NGS datasets that are now being routinely generated. TrawlerWeb is freely available and accessible at: http://trawler.erc.monash.edu.au.

**Electronic supplementary material:**

The online version of this article (10.1186/s12864-018-4630-0) contains supplementary material, which is available to authorized users.

## Background

Understanding how genes are regulated is the challenge of the post-genomic era [1–4]. The ability to identify *cis*-regulatory combinations [5, 6] that affect the spatiotemporal control of gene expression is important in elucidating disease and developmental processes [7, 8]. Genome-wide binding assays such as chromatin immunoprecipitation (ChIP) [9], DNA adenine methyltransferase identification (DamID) [10, 11], and transposase-accessible chromatin (ATAC) [12] coupled with next-generation sequencing (NGS) technologies are popular methods to uncover regulatory regions that will shed light on our understanding of gene regulation [13, 14]. These methods have been co-opted amongst scientists working on model organisms ranging from plants to humans [15, 16]. The downstream analysis of these genome-wide assays consists of identifying specific binding motifs in DNA, which ultimately encode for regulatory information. To achieve this, several motif discovery algorithms have been generated to identify specific binding motifs using different algorithms, such as exhaustive pattern-based enumeration, variants of Markov chain Monte Carlo (MCMC) or deep learning models [17–23].

As NGS technologies are now routinely used in all laboratories, with the emergence of more online platforms for NGS data processing e.g. Galaxy [24], ChIP-seq tools [25], there is a need to streamline the motif discovery process, ideally with the convenience of web-based interfaces accepting input queries. Accurate binding site prediction, speed and ease-of-use are key criteria for users when selecting a preferred motif discovery tool. Finally, selecting the motifs to be experimentally tested amongst the list of predicted motifs still represents a challenge.

Two of the most cited tools for *de novo* motif discovery, MEME-ChIP and RSAT peak-motifs provide a user-friendly interface and have been used to successfully identify transcription factor binding sites [18, 20]. DeepSEA also offers an online web search interface, but input sequences are currently limited to 1000 base pairs (bp) and only queries against the Human Genome (hg19) [22]. Trawler_standalone is one of the fastest motif discovery tools available, while still providing accurate predictions [5], however it is currently only available as a command-line standalone version [6]. Here we present TrawlerWeb, which streamlines *de novo* motif discovery with NGS datasets from a wide range of species. This web-based version provides three new unique features that allow it to streamline and facilitate the analysis of predicted motifs: 1) in addition to FASTA-formatted sequences, it accepts direct input from ChIP-seq experiments in BED format, 2) it automatically generates a set of background sequences matching the input sequences in terms of genomic location and 3) it allows the ranking of predicted motifs by conservation score to select those more suited for downstream experimental validation. After systematically comparing TrawlerWeb with the web versions of MEME-ChIP and RSAT peak-motifs, we demonstrated that in accordance with Trawler_standalone performance [5], TrawlerWeb still remains the fastest online motif discovery tool while maintaining motif prediction accuracy.

## Implementation

### Web implementation

TrawlerWeb is running on a standard Apache web server configuration under a Linux environment. It has been deployed and supported on the Monash node (R@CMon) of the Nectar Research Cloud. TrawlerWeb has been rigorously tested by a total of 11 different users on five different datasets using Chrome, Firefox and Internet Explorer web browsers with Linux, Windows and Mac OS X operating systems (Table 1).

**Table 1:** Operating systems and browsers on which 11 users have successfully tested TrawlerWeb

User	Operating System	Used browser
001	MAC OS X 10.11	Mozilla Firefox
002	Windows 10	Google Chrome
003	MAC OS X 10.11.6	Mozilla Firefox
004	Windows 8.1	Google Chrome
005	MAC OS X 10.10	Mozilla Firefox
006	Linux Ubuntu 16.04	Mozilla Firefox
007	Windows 7 Enterprise	Mozilla Firefox
008	MAC OS X 10.11	Google Chrome
009	MAC OS X 10.9.5	Google Chrome
010	Windows 7 Enterprise	Internet Explorer
011	MAC OS X 10.9.5	Google Chrome

### Input file

The web interface for TrawlerWeb offers users the option of uploading input sequences as BED indexed format, in addition to FASTA files, which is the most common form of input offered by most motif discovery tools. BED files are lists of genomic intervals and are the direct output from ChIP-seq experiments. Users can therefore directly input the data of their ChIP-seq experiments in TrawlerWeb, without having to retrieve corresponding FASTA sequences. Note that PSCAN [21] also offers BED file input but performs motif discovery on known motifs rather than *de novo*. DeepSEA [22] accepts input files as BED format, however, currently only one genome (hg19) is supported and input sequence length is limited to 1000 bp. Since peaks obtained from ChIP-seq on histone modifications, for example, could exceed 1000 bp, this option might be restrictive to users. At present, TrawlerWeb remains the only online motif discovery tool accepting input files in BED format for a large range of species.

Regions can be either uploaded as BED files or directly pasted into the website, with users required to select the genome assembly of their desired model organism. To date, TrawlerWeb supports 16 genome assemblies (Table 2), downloaded from EnsEMBL [26]. To streamline analysis of data generated from ChIP-seq experiments and other DNA-binding assays, FASTA sequences corresponding to the input BED regions are automatically extracted from locally stored chromosome assemblies. Prior to retrieving FASTA sequences, the BED regions are processed using BEDtools’ merge tool with default settings to avoid duplicated locations [27]. FASTA sequences are repeat-masked to prevent the discovery of repetitive motifs produced from low-complexity and repeat regions [5]. Users also have the option of using non-masked sequences if the immunoprecipitated transcription factor is known or hypothesised to bind to repeat sequences [28].

**Table 2:** Species and genome assemblies supported by TrawlerWeb

	Species	Genome assemblies
Fish		
Medaka	*Oryzias latipes*	oryLat2
Zebrafish	*Danio rerio*	danRer7
Stickleback	*Gasterosteus aculeatus*	gasAcu1
Tetrapods		
Human	*Homo sapiens*	hg19, hg38
Mouse	*Mus musculus*	mm9, mm10
Rat	*Rattus norvegicus*	rn5
Marmoset	*Callithrix jacchus*	calJac3
Chicken	*Gallus gallus*	galGal3
Clawed frog	*Xenopus tropicalis*	xenTro3
Other eukaryotes		
Fruit fly	*Drosophila melanogaster*	dm3, dm6
Worm	*Caenorhabditis elegans*	ce10
Yeast	*Saccharomyces cerevisiae*	sacCer3
Thale cress	*Arabidopsis thaliana*	TAIR9

### Background file

For each set of input BED regions, a randomised background specific to the dataset is automatically generated to match the distribution of the genomic locations of the input regions. This input-matched background allows TrawlerWeb to account for sequence biases present in certain genomic regions (for example promoter regions are known to be enriched in CpG islands [29]).

To generate a customised background dataset, first, TrawlerWeb calculates the distribution of the distances of the input regions with respect to the nearest transcription start site (TSS). Each input region is associated to a ‘nearest gene’ and the distance of this region to the gene’s TSS is calculated using gene coordinates downloaded from EnsEMBL using BioMART [26, 30] for the given organism. Distances are then plotted across discrete ranges (e.g. -5000 bp to 0, 0 to 5000 bp, etc.) to produce a frequency table representing the input regions. Next, genes are randomly selected from the entire genome and genomic regions are extracted upstream or downstream of the TSS, so that the distribution of the selected regions match the frequency table generated for input regions. This frequency table is displayed on the results page as TrawlerWeb is running. The amount of randomly selected regions to generate this background dataset is eight times the amount of regions in the input, which we have previously demonstrated to robustly provide adequate background dataset [5, 6].

As a new background will be generated for each new input submission, users have the option to download the background FASTA sequences generated for a specific FASTA input, should they need to re-run the exact same analysis using the same background.

### Input options

TrawlerWeb comes with an array of options to optimise the user’s search results. By default, Trawler will search for motifs that are at least 8 bp in length and at most 20 bp. However, the user can reduce the minimum motif length in order to allow motifs of shorter length to be identified.

The wildcard option allows for mismatches in the identified motif. For a minimum motif length of 8, two wildcards are used by default. Should the user choose to reduce the minimum motif length (e.g. 6), one wildcard is recommended to maintain sensitivity.

If the final list of clustered motifs (named families) in the results page retain some similarity and should be clustered together, the “percentage overlap” option can be reduced so that the amount of similarity required between instances to be clustered is reduced.

The frequency at which a motif is expected to occur in a sample can vary depending on the type of data. Generally, a minimum of 10 to 20 occurrences is suitable for most ChIP experiments. If this parameter is set too low, Trawler will be unable to identify any significant results whereas setting the parameter too high when the desired motif is not present in all sample sequences will produce a motif with low information content. This can be configured using the “occurrence” option.

The number of motifs used for clustering is determined by the “number of motifs” option. By default, Trawler takes the top 200 ranked motifs for clustering. To allow for identification of secondary and possibly tertiary motifs, the number of motifs can be increased, however this will also increase computation time.

After running Trawler initially with default settings once, the final number of motifs identified can be fine-tuned by indicating a value for “number of clusters” option. By default, Trawler will cluster by strongly connected component (SCC) when “number of clusters” is set to zero. However, k-means clustering can be used to cluster the motifs into n clusters by providing an integer value (n) to this option.

### Output files and data download

The final results are summarised as a web page displaying all discovered motifs in a table along with z-scores for statistical over-representation [5]. Putative matches against known TFBSs and maximum sequence conservation of the motif’s instances will be displayed in this summary web page. By default, motifs are ordered in decreasing order of conservation score, however any of the columns can be used for sorting the output. For each identified motif, further information is available upon clicking on the motif name of logo. First, the distribution of the motif locations within the input sequences can be visualised as a histogram. Consensus sequence, length and identity of the TFBS match are displayed in the next table where a mouse-over on the column titles will provide a description of the properties of the match. A link to the original TFBS will allow to visualise the putative hit from either UniPROBE, Jaspar and HOCOMOCO databases [31–34]. The final table lists the location of every instance of the predicted motif in the input sequences, along with an average and maximum conservation score within the instance. Each location is linked to a dynamic view of the region in the UCSC genome browser [35]. Similarly, a mouse-over on the column’s title will provide further information. All tables in this detailed page are sortable by column, searchable and are dynamically filtered for the searched term. The entire results webpage and files generated by TrawlerWeb are also available for download in a single zip file.

## Results and discussion

### TrawlerWeb runs the fastest amongst popular web-based motif discovery tools

We aimed to compare the performance of TrawlerWeb with the popular web-based *de novo* motif discovery tools RSAT peak-motifs [20, 36] and MEME-ChIP [18]. For this, 11 users were given five different ChIP-seq datasets from five commonly used model organisms in FASTA format (Table 3). The same FASTA input file was used across the three different programs with the same background file used for TrawlerWeb and RSAT peak-motifs, default background was used for MEME-ChIP as it does not allow for custom background FASTA input. All other options were kept as default. Running time was recorded from when the “Submit Query”, “GO” or “Start Search” button was clicked for TrawlerWeb, RSAT peak-motifs and MEME-ChIP respectively, until the final list of motifs was returned. This also includes the queuing time which realistically reflects the actual waiting time experienced by users.

**Table 3:** ChIP-seq on transcription factors and genome assemblies used to compare TrawlerWeb, RSAT peak-motifs and MEME-ChIP

Transcription factor	ChIP-seq GEO accession number	Reference for ChIP-seq	ChIP-seq dataset size (kbp)	Reference for known binding site	Species	Genome
Zic3.2	GSM1017643	Winata et al., 2013 [49]	282	JASPAR PB0207.1	*D. rerio*	danRer7
TOC1	GSM878068	Huang et al., 2012 [47]	343	Huang et al., 2012	*A. thaliana*	TAIR9
MEF2A	GSM1377538	Houles et al., 2015 [46]	338	JASPAR MA0052.3	*M. musculus*	mm9
Su(H)	GSE66225	Skalska et al., 2015 [48]	475	JASPAR MA0085.1	*D. melano-gaster*	dm3
Sox15.1	GSM1536045	Sulahian et al., 2015 [40]	1783	JASPAR PB0065.1	*H. sapiens*	hg19

**Fig. 1:**
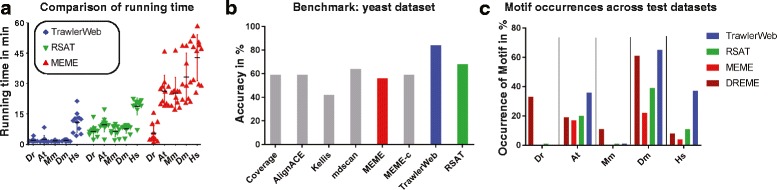
Comparing the performance of TrawlerWeb with other web-based motif discovery tools. **a** Duration of 11 independent runs for TrawlerWeb (blue), RSAT peak-motifs (green) and MEME-ChIP (red) in minutes. The mean is represented by the horizontal line for each dataset. The error bars indicate the standard deviation from the mean. The data are ordered by increasing size of the FASTA input file from left to right. Note that MEME-ChIP did not find any motifs for *Dr*, hence the motif discovered by DREME was used (see also Fig. 2a). **b** Overall performance benchmark of TrawlerWeb against 7 other algorithms, using 65 ChIP pulled down experiments on yeast dataset from [38]. MEME-c: MEME algorithm run on conserved regions only. **c** Comparison of percentage occurrence of over-represented motifs across test datasets. Motif discovery were conducted using 4 algorithms (DREME, MEME, RSAT peak-motifs, and TrawlerWeb) on the test datasets and the number of sequences containing the highest scoring motif were expressed as a percentage of the total number of analysed input sequences. The MEME-ChIP pipeline uses both MEME and DREME motif discovery tools for finding relatively long and short motifs respectively. The MEME algorithm uses a random subsample of 600 sequences. *Dr* = *Danio rerio*, *At* = *Arabidopsis thaliana*, *Mm* = *Mus musculus*, *Dm* = *Drosophila melanogaster*, *Hs* = *Homo sapiens*

For all five species tested (Table 3), TrawlerWeb ran the fastest (Fig. 1a) in accordance with its standalone version [5, 6]. TrawlerWeb generally returned the discovered motifs in less than 2 min (min) for the four smaller datasets *Danio rerio* (*Dr*), *Arabidopsis thaliana* (*At*), *Mus musculus* (*Mm*) and *Drosophila melanogaster* (*Dm*). Only for the human dataset (*Hs*), being the largest input file tested, running time ranged from 4 min to over 21 min, averaging 10 min overall. RSAT peak-motifs identified motifs after 2-10 min with only a few outliers. Motif discovery for the human dataset ran for 17 to 22 min, however, one run was completed in under 7 min. Nonetheless, we experienced larger variations in processing times with RSAT peak-motifs compared to TrawlerWeb. MEME-ChIP had the longest running time among the three tested tools. Typical motif discovery was completed on average after about 30 min, with the exception of *Hs* which finished after up to almost 1 h. Of note, MEME-ChIP provides by default two different algorithms, MEME and DREME. DREME [37] discovers short and ungapped motifs. Since MEME did not find any motifs for the zebrafish dataset (*Dr*) we used the results provided by DREME, which can explain the shorter time compared to the performance of MEME.

In conclusion, in all tested cases, TrawlerWeb outperformed popular web-based *de novo* motif discovery tools in terms of speed.

### TrawlerWeb accurately identifies the expected binding site

To measure TrawlerWeb’s accuracy against other online motif discovery tools, we tested TrawlerWeb against a yeast ChIP benchmark dataset [38]. This same dataset was previously used to test Trawler_standalone algorithm against other software [5, 6] but lacked RSAT peak-motifs [20], which we have included in this run. On this yeast dataset, TrawlerWeb outperformed all other methods in terms of accuracy, identifying 54 out of 65 motifs previously found (85%; Fig. 1b and Additional file 1: Table S1). These results were expected since TrawlerWeb and Trawler_standalone share the same underlying algorithm.

**Fig. 2:**
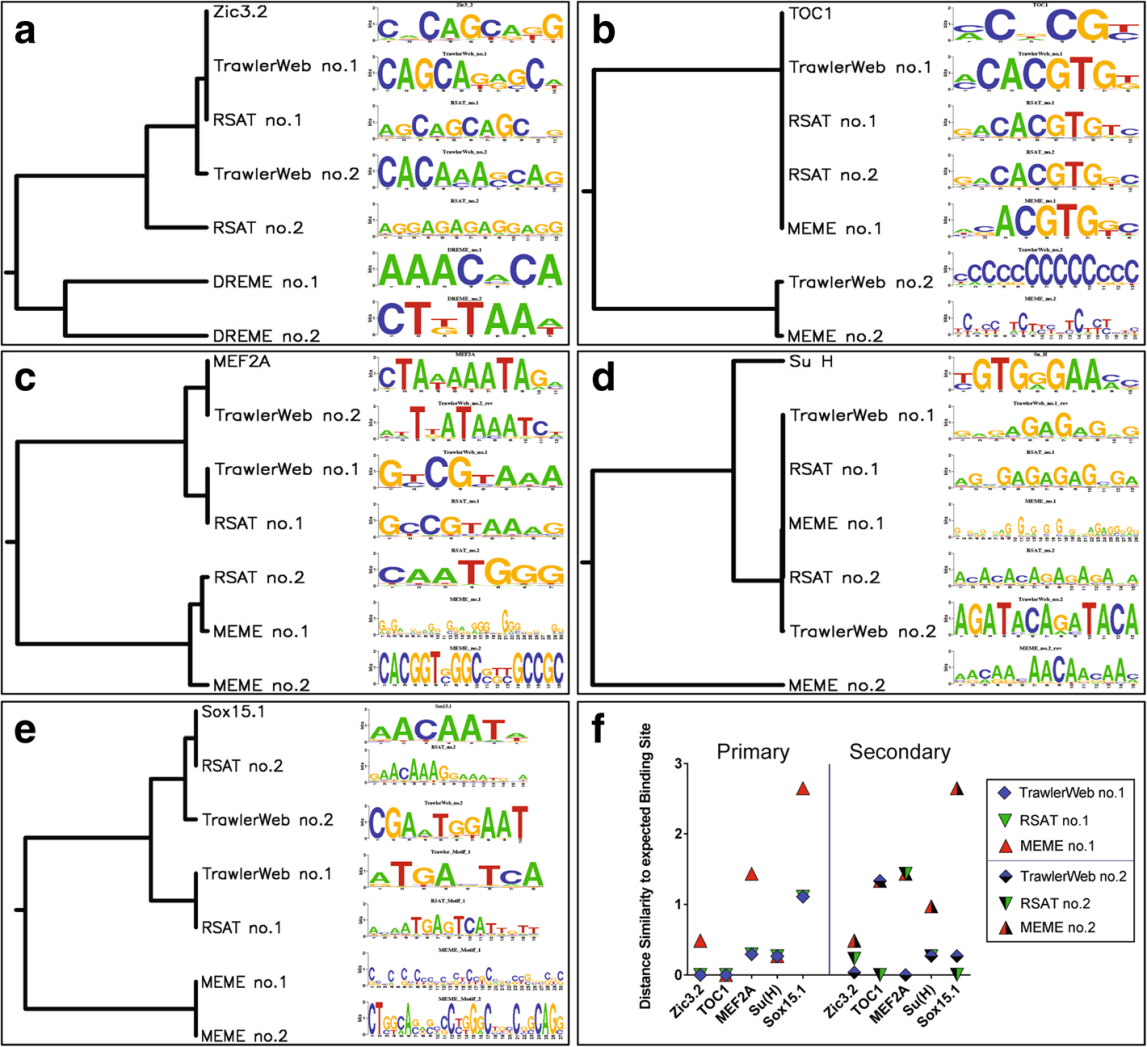
Finding the expected motif with TrawlerWeb, RSAT peak-motifs, and MEME-ChIP. Alignment of the closest primary (no.1) and secondary (no.2) motif to the expected binding site identified for each motif discovery tool for the five species **a** *Danio rerio*, **b** *Arabidopsis thaliana*, **c** *Mus musculus*, **d** *Drosophila melanogaster*, and **e** *Homo sapiens*. **f** For each tool, Similarity Distance of the closest primary (no.1) and secondary (no.2) motif to the expected binding site. Motifs of 6 nucleotides (nt) length were represented for Su(H) and Sox15.1, and 7 nt for MEF2A, TOC1, and Zic3.2. MEME did not find any motif for Zic3.2, motif found by DREME was used

In order to further characterise TrawlerWeb’s accuracy to identify the expected binding sites, we compared the motifs predicted by each tool, across eight users, to the known binding site of the transcription factor of interest, for the five ChIP-seq datasets (Table 3). To identify which predicted motif best matched the known binding site, all predicted motifs with the two highest z-scores (TrawlerWeb) or lowest e-values (RSAT peak-motifs, best of 6 nucleotide or 7 nucleotide length, and MEME/DREME) were aligned and clustered with the expected binding sites (Table 3) using STAMP (default options) [39]. For each ChIP-seq dataset, and for each program, motifs with the shortest distance to the expected binding site were identified as the closest motif. Across all users, the closest primary and secondary motifs (Fig. 2a-e), and corresponding distance to the expected binding site (Fig. 2f), were downloaded from STAMP in Newick format and visualised with iTOL using the expected motif as a reference. In two cases out of five, TrawlerWeb and RSAT predicted the known binding sites (for Zic3.2 (Fig. 2a), TOC1 (Fig. 2b)). TrawlerWeb is the only tested tool that identified the expected binding site for MEF2A (Fig. 2c), albeit as a secondary motif. In the case of Su(H) (Fig. 2d), TrawlerWeb and RSAT peak-motifs identified the same primary motif, which interestingly, is different to the expected binding site. Finally for Sox15.1 (Fig. 2e), RSAT peak-motifs discovered a similar motif to the known binding site, whereas TrawlerWeb found motifs that are quite different. However, these motifs resemble the PWM of Sox15.2 [32] suggesting that the reported binding site of Sox15 [40] is Sox15.2 rather than Sox15.1. In two out of five cases (Fig. 1b, d) the primary motifs discovered by MEME-ChIP agreed with the other motif discovery tools and the expected binding site. For MEF2A and Sox15.1, the motifs identified by MEME-ChIP have low similarity to the known binding sites. For Zic3.2 MEME-ChIP did not find any motifs, hence we used DREME, which found two motifs that are quite distant from the expected binding site.

Of note, in three out of five datasets, TrawlerWeb identifies the predicted motif from the highest number of input sequences. When comparing over-represented motifs (based on the percentage of motif occurrences of the highest score) discovered using DREME, MEME, RSAT peak-motifs, and TrawlerWeb (Fig. 1c), TrawlerWeb returns the highest percentage occurrence in *dm3*, *hg19* and *tair9* test datasets (65.5%, 37.6% and 36.6% respectively), and delivers occurrences comparable to both MEME and RSAT peak-motifs in the *mm9* and *zv9* datasets (1.6% and 0.3%) (Additional file 2: Table S2). Variations in motif occurrence could be due to the presence of a secondary motif corresponding to a co-factor of the TF of interest [11]. Indeed, for TOC1, MEF2A and Su(H) datasets, TrawlerWeb identified a motif different to the known binding site (Fig. 2b-d). Overall, TrawlerWeb robustly identifies the primary motif with high similarity to the expected binding site (Fig. 2f).

### TrawlerWeb offers the unique option of displaying motif conservation scores

**Fig. 3:**
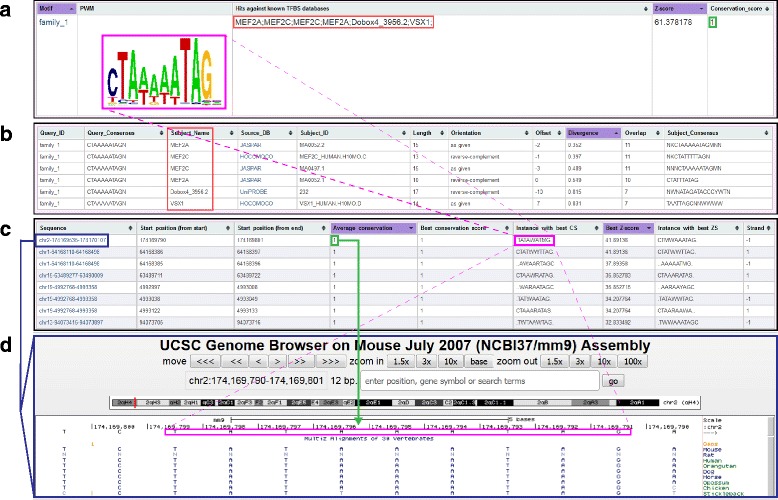
TrawlerWeb output display with conservation scores and UCSC links. **a** TrawlerWeb displays the Position Weight Matrices (PWMs, pink box), Hits against known transcription factor binding site (TFBS) databases (red box), Z-scores of the discovered motifs, and the Conservation Score (green box). **b** Clicking on the PWM (pink box in (**a**)) directs the user to the list of putative matches (red box) and provides a direct link to the corresponding TFBS database entry. **c** Chromosomal positions of instances of the discovered motif (pink box) in the input peaks are also provided. Average and maximum conservation score (green box) will be available for each instance of the PWM. Clicking on the genomic region of interest (blue box) opens it in the UCSC Genome Browser (**d**)

Motif discovery tools deliver a list of over-represented putative DNA binding sites, usually ranked by over-representation score. Motifs are then often selected for experimental validation, for instance by verifying whether the transcription factor of interest effectively binds to the predicted motif. Amongst all of the instances of the predicted binding site in the submitted sequences, selecting the ones for experimental validation is not trivial. In order to prioritise identified motifs for downstream analysis, evolutionary conservation has been used as a proxy to select for the binding sites which are likely to be functional. Indeed, TFBSs harbouring an essential function are under strong evolutionary constraint compared to neutrally evolving non-coding sequences, and will therefore display higher sequence conservation [41]. We have implemented this feature in TrawlerWeb whereby when input files are provided in BED format, corresponding genomic coordinates are matched against the reference genome selected. This provides the unique advantage for TrawlerWeb over other tools to display conservation scores for every instance of motifs discovered. To display conservation scores, PhastCons scores were downloaded from UCSC [35] and stored along with the chromosome sequences for each organism. For each instance of the identified motif in the input sequences, the average and maximum conservation score is calculated using the “bigWigOverAverage” tool provided in kentUtils [42] package from UCSC. Version 305 of kentUtils source is used as it offers “minMax” option to calculate the maximum value for conservation. The average and maximum conservation scores calculated from PhastCons scores are automatically displayed in the output (Fig. 3). Although this option is only available with the BED input option, it provides scientists the opportunity to filter for the evolutionarily conserved predicted binding sites for downstream biological validations.

## Conclusions

Downstream analyses of DNA-binding genome-wide assays are paramount in elucidating a precise binding motif and in addition, identifying secondary proximal binding partners. As these experimental protocols become widely used, there is a need for easy access tools for scientists from all fields of research to perform *de novo* motif discovery. Here we have developed TrawlerWeb to allow fast and streamlined *de novo* motif discovery online, allowing direct search from NGS outputs using the BED format and generating an input-matched background. We have shown that TrawlerWeb performs faster than most popular motifs discovery tools, and robustly identifies the expected binding site. TrawlerWeb is primarily used to identify over-represented motifs in regions of DNA in ChIP-seq experiments for both transcription factors and histone marks. However, it can also be extended for identification of microRNA targets [6, 43], RNA-binding protein targets [44] or co-expression groups [45].

In conclusion, TrawlerWeb will appeal to a wide range of fields as the breadth of genome assemblies supported include commonly studied model organisms (Table 2). However, this list is currently limited to organisms for which conservation scores are available. To circumvent the need of a conservation score, users are still provided with the option to use FASTA formatted files with TrawlerWeb. Using FASTA input expands the range of analysis that can be performed with TrawlerWeb, by permitting analysis of datasets from partially sequenced genomes and from non-model organisms.

## Availability and requirements

**Project name:** TrawlerWeb.

**Project home page:** https://trawler.erc.monash.edu.au/

**Operating system(s):** Platform independent.

**Programming language:** Java, Perl, HTML.

**Other requirements:** N/A.

**License:** The GNU General Public *License* (GPL) for Trawler_standalone.

**Any restrictions to use by non-academics:** N/A.

## Additional files

Additional file 1: **Table S1.** Details of the assessment of TrawlerWeb. Detailed table of Fig. 1b showing, for each ChIP experiment, the ability of individual programs to uncover the correct binding site in yeast. For each individual ChIP experiment, the success or failure of 8 different algorithms including TrawlerWeb is shown. The results from the 6 algorithms (Coverage, AlignACE, Kellis, mdscan, MEME, and MEME-c) were extracted from Harbison et al. 2004 [38]. The matching motifs found by TrawlerWeb are identical to that found by Trawler_standalone (detailed previously in Ettwiller et al. 2007 [5]). The results from RSAT were performed by this study, where the matching motifs found by RSAT were described in the last column. (XLSX 190 kb)

Additional file 2: **Table S2.** Details of motif occurrence comparison between web-based motif discovery software. The highest scoring motifs discovered in DREME, MEME, RSAT peak-motifs, and TrawlerWeb and their corresponding occurrences are illustrated here. For the highest scoring motif, the number of motif occurrences were expressed as a percentage of the total number of input sequences. *MEME-ChIP pre-processes submitted sequences longer than 100 by trimming them evenly from both ends to get the centered 100 bp sequence and discards trimmed sequences containing only Ns from repeat masking. **MEME motif discovery automatically limits the run to a randomly sampled 600 sequences to reduce run time. (XLSX 576 kb)

## Abbreviations

ATAC: Assay for transposase-accessible chromatin
ChIP-seq: Chromatin immunoprecipitation followed by high-throughput sequencing
DamID: DNA Adenine methyltransferase identification
DREME: Discriminative regular expression motif elicitation
MCMC: Markov chain Monte Carlo
NGS: Next-generation sequencing
TFBS: Transcription factor binding site
